# Lumbago

**Published:** 1892-06

**Authors:** R. A. Chudleigh


### LUMBAGO.

This complaint is simply lumbar rheumatism, or a rheumatic inflammation of the muscles of the loins, and its treatment does not materially differ from that of any other local rheumatism. The querist's wishes with regard to drugs are met half-way already by the fact that opinions are not concordant in favor of any particular drug in dealing with this disorder. In the first place try to avoid an attack of lumbago by keeping the loins protected and supported by a broad and thick flannel belt, by never remaining in wet and cold clothes, and by avoiding muscular over strain, especially such as bears directly on the lumbar muscles.

Hæmorrhoidal congestion produces something indistinguishable from lumbago, which disappears almost immediately on the development of the hæmorrhoids. In this last case the treatment must be addressed to the liver, and the (portal) circulation specially connected with it. As inflammation of the spine may ensue, this kind of lumbar pain should not be neglected. When once an attack of lumbago has begun, it is essential to give entire rest to the affected muscles. A very slight exertion of them seems to tear the tissue, and aggravates the pain excessively. Lie on the back with a small pillow in the hollow of the loins, and when it is necessary to rise, be helped up.

All liniments are useful; and so is massage, which should be performed with the edge of the hand, while the patient lies on his face. The hand must be placed at the lower and outer limit of the painful region, and be passed steadily up and in, to the line of the spine; repeat the strokes, with both hands at once, or with one at a time, from twenty to one hundred times, as the symptoms indicate. "Cave in" to lumbago at once, and do not attempt to brave it out or work it off. Such a thing may be successfully done in a very light attack ; but the experiment is a very hazardous one. As to diet, abstain from alcohol and the well known list of inflammatory and over-stimulating foods and beverages.

There is a caution of very great importance, namely, when you have lumbar pains make sure that they are lumbago before you treat them as such. For lumbar pains may arise from inflammation of the kidneys or spinal caries, or disease of the aorta, or internal abscess, or sundry other affections, which I need not specify.—R. A. CHUDLEIGH, M. D.

